# Identification of Cyclophilin A as a Potential Anticancer Target of Novel Nargenicin A1 Analog in AGS Gastric Cancer Cells

**DOI:** 10.3390/ijms22052473

**Published:** 2021-03-01

**Authors:** Jang Mi Han, Jae Kyung Sohng, Woo-Haeng Lee, Tae-Jin Oh, Hye Jin Jung

**Affiliations:** 1 Department of Life Science and Biochemical Engineering, Sun Moon University, Asan 31460, Korea; gkswkdal200@naver.com (J.M.H.); sohng@sunmoon.ac.kr (J.K.S.); saint832@sunmoon.ac.kr (W.-H.L.); tjoh3782@sunmoon.ac.kr (T.-J.O.); 2 Department of Pharmaceutical Engineering and Biotechnology, Sun Moon University, Asan 31460, Korea; 3 Genome-Based BioIT Convergence Institute, Asan 31460, Korea

**Keywords:** nargenicin A1 analog, compound 9, cyclophilin A, CD147, gastric cancer

## Abstract

We recently discovered a novel nargenicin A1 analog, 23-demethyl 8,13-deoxynargenicin (compound 9), with potential anti-cancer and anti-angiogenic activities against human gastric adenocarcinoma (AGS) cells. To identify the key molecular targets of compound 9, that are responsible for its biological activities, the changes in proteome expression in AGS cells following compound 9 treatment were analyzed using two-dimensional gel electrophoresis (2-DE), followed by MALDI/TOF/MS. Analyses using chemical proteomics and western blotting revealed that compound 9 treatment significantly suppressed the expression of cyclophilin A (CypA), a member of the immunophilin family. Furthermore, compound 9 downregulated CD147-mediated mitogen-activated protein kinase (MAPK) signaling pathway, including c-Jun N-terminal kinase (JNK) and extracellular signal-regulated protein kinase 1/2 (ERK1/2) by inhibiting the expression of CD147, the cellular receptor of CypA. Notably, the responses of AGS cells to CypA knockdown were significantly correlated with the anticancer and antiangiogenic effects of compound 9. CypA siRNAs reduced the expression of CD147 and phosphorylation of JNK and ERK1/2. In addition, the suppressive effects of CypA siRNAs on proliferation, migration, invasion, and angiogenesis induction of AGS cells were associated with G2/M cell cycle arrest, caspase-mediated apoptosis, inhibition of MMP-9 and MMP-2 expression, inactivation of PI3K/AKT/mTOR pathway, and inhibition of hypoxia-inducible factor-1α (HIF-1α) and vascular endothelial growth factor (VEGF) expression. The specific interaction between compound 9 and CypA was also confirmed using the drug affinity responsive target stability (DARTS) and cellular thermal shift assay (CETSA) approaches. Moreover, in silico docking analysis revealed that the structure of compound 9 was a good fit for the cyclosporin A binding cavity of CypA. Collectively, these findings provide a novel molecular basis for compound 9-mediated suppression of gastric cancer progression through the targeting of CypA.

## 1. Introduction

Gastric cancer (GC) is a malignant tumor that originates from the glandular epithelium of the gastric mucosa. GC ranks fifth in incidence and third in mortality worldwide, thereby making it a serious public health concern [1]. The incidence rate of GC is markedly high in East Asia, including China and Korea, whereas it is relatively low in North America and Northern Europe [1]. GC is a multifactorial disease caused by diverse environmental and genetic factors. Risk factors, such as family history, diet, alcohol consumption, smoking, obesity, *Helicobacter pylori* infection, E-cadherin gene (*CDH1*) alteration, and polymorphisms of interleukin (IL-17 and IL-10) genes, are associated with a high incidence of GC [2,3,4,5]. As noticeable symptoms do not occur until the GC reaches an advanced stage, late diagnosis contributes to a poor prognosis with limited treatment options [6]. Unfortunately, most of the known biomarkers for GC diagnosis and prognosis have low sensitivity and specificity [6]. Chemotherapy, radiation therapy, and gastrectomy have been recognized as major treatment modalities for GC, but they generally cause serious side effects or have limited success rates [7,8]. The standard chemotherapy for advanced GC includes a combination of fluoropyrimidine and platinum compounds, with human epidermal growth factor receptor 2 (HER2)-targeted trastuzumab, vascular endothelial growth factor receptor 2 (VEGFR2)-targeted ramucirumab, and immune checkpoint inhibitors, nivolumab and pembrolizumab [9]. Despite multimodal treatment, the prognosis for advanced GC remains poor due to complex oncogenic molecular mechanisms caused by various genetic and epigenetic alterations [10]. These GC therapies, not only cause symptoms, such as fatigue, diarrhea, and rash, but also cause fatal side effects, such as anemia, intestinal obstruction, cardiac dysfunction, and blood toxicity [9]. In addition, drug resistance remains a major challenge in GC therapy [11]. Therefore, safer and more effective targeted anticancer agents need to be developed to improve the treatment of incurable GC. 

Natural microbial products and their derivatives have been recognized as sources of therapeutic agents and of structural diversity [12]. Nargenicin A1 is a major secondary metabolite produced by *Nocardia* species and has effective antibacterial activity against various gram-positive pathogenic bacteria [13]. In our recent study, we characterized the tailoring steps for the biosynthesis of nargenicin A1 in *Nocardia* sp. CS682 which resulted in the generation of several new analogs of the natural product [14]. Analysis of the bioactivity of the analogs revealed that 23-demethyl 8,13-deoxynargenicin (compound 9) possesses potential antitumor activity, unlike nargenicin A1 and the other analogs. Compound 9 suppressed the growth of various cancer cell lines, including gastric, lung, skin, liver, colon, brain, breast, and cervical cancer, within a range of concentrations that did not affect normal cell growth [14]. Notably, compound 9 showed the highest inhibitory effect on the proliferation of GC cells. Further analysis of the underlying molecular mechanism of its anticancer effect demonstrated that compound 9 exhibits a growth inhibitory effect against AGS human GC cells by inducing G2/M cell cycle arrest and reactive oxygen species (ROS)- and caspase-mediated apoptosis through the downregulation of the phosphatidylinositol 3-kinase (PI3K)/AKT/mammalian target of rapamycin (mTOR) pathway [14]. More recently, we found that compound 9 exerts antiangiogenic activity via dual downregulation of vascular endothelial growth factor (VEGF)/VEGFR2-mediated signaling in human umbilical vein endothelial cells (HUVECs) and hypoxia-inducible factor-1α (HIF-1α)/VEGF expression in AGS GC cells [15]. 

In the current study, we identified for the first time the primary cellular target of compound 9 to improve our understanding of the molecular mechanisms involved in its anticancer and antiangiogenic activities against GC cells. The changes in proteome expression mediated by compound 9 treatment of AGS cells were analyzed using two-dimensional gel electrophoresis (2-DE), followed by matrix assisted laser desorption ionization-time of flight mass spectrometry (MALDI-TOF MS). Proteomic analysis and further functional experiments revealed that the anticancer and antiangiogenic effects of compound 9 against GC cells are associated with the downregulation of cyclophilin A (CypA) function. In addition, the biological activity of compound 9 was significantly correlated with the response to CypA knockdown in AGS cells. Furthermore, the results of drug affinity responsive target stability (DARTS), cellular thermal shift assay (CETSA), and docking modeling approaches revealed that compound 9 binds to CypA. Therefore, this study suggests that compound 9 is a new anticancer agent that targets CypA, which plays a key role in GC development.

## 2. Results

### 2.1. 2-DE Profiling of the Differentially Expressed Proteins in AGS Cells Following Treatment with Compound 9

**Figure 1:**
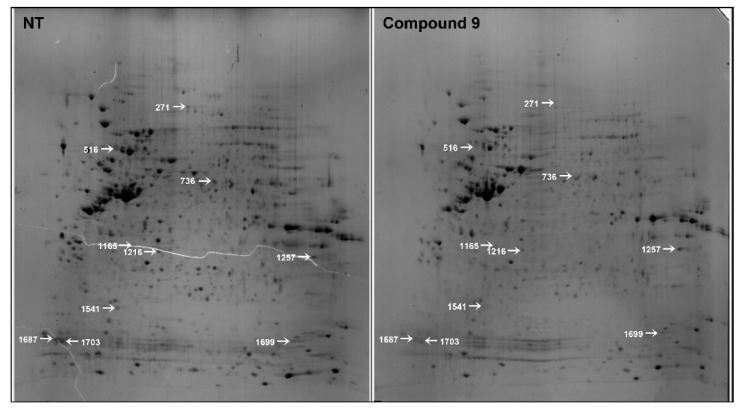
Two-dimensional gel images of AGS cells treated with or without compound 9. Total protein extracts were separated on pH 3.0–10.0 nonlinear IPG strips in the first dimension followed by 12% SDS-PAGE in the second dimension and visualized by CBB staining. Ten protein spots that were downregulated more than 3-fold by compound 9 (400 μM) treatment were identified using MALDI-TOF MS analysis. The selected spots are marked with white arrows.

Chemical proteomic approaches are used to assess the interactions between drugs and therapeutic target proteins [16]. To identify the anticancer target of compound 9 in GC cells, the changes in protein profile in AGS cells in response to compound 9 treatment were investigated using proteomic analysis, including 2-DE and MALDI-TOF MS. Proteins extracted from either untreated (control) or compound 9-treated AGS cells were resolved by 2-DE and the stained 2D gel images were analyzed using Delta 2D software (Figure 1). A total of 1032 and 840 protein spots were detected in the control, and compound 9-treated gels, respectively. Among the protein spots that were differentially expressed between the control and compound 9-treated cells, ten protein spots that were downregulated more than 3-fold by compound 9 treatment were identified using MALDI-TOF MS analysis and database searching. The ten identified proteins are listed in Table 1.

**Figure 2:**
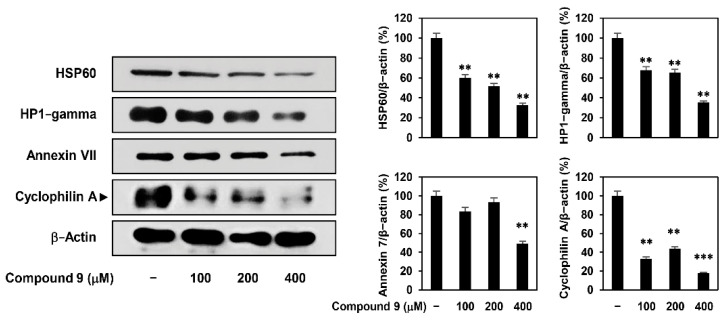
Validation of the identified protein spots. AGS cells were treated with compound 9 (100–400 μM) for 24 h. Protein levels were detected by western blot analysis and further quantified by densitometry. The level of β-actin was used as an internal control. ** *p* < 0.01, *** *p* < 0.001 vs. the control.

To validate the data obtained from 2-DE, the changes in protein expression in AGS cells were analyzed by western blotting. As shown in Figure 2, heat shock protein 60, HP1-gamma, annexin VII, and CypA were downregulated following compound 9 treatment, which was consistent with the 2-DE results. Compound 9 treatment markedly suppressed the expression of CypA.

### 2.2. Effect of Compound 9 on CypA/CD147 Pathway in AGS Cells

**Figure 3:**
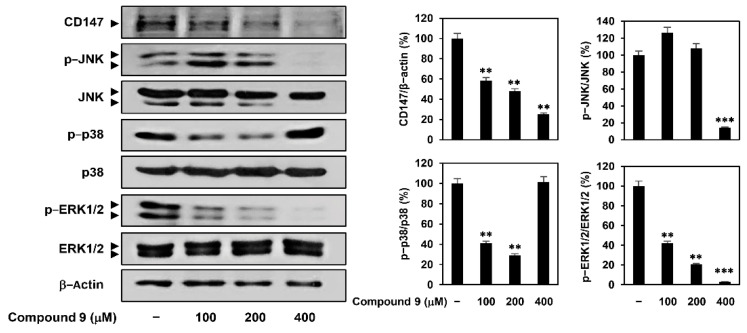
Effect of compound 9 on the CypA/CD147 pathway in AGS cells. AGS cells were treated with compound 9 (100–400 μM) for 24 h. Protein levels were detected by western blot analysis and further quantified by densitometry. The level of β-actin was used as an internal control. ** *p* < 0.01, *** *p* < 0.001 vs. the control.

CypA is a member of the immunophilin family with peptidyl prolyl cis-trans isomerase activity and plays important roles in protein folding, trafficking, assembly, immune-modulation, and cell signaling [17,18]. Several studies have demonstrated the overexpression of CypA and its cellular receptor CD147 in many malignant tumors including GC [19,20]. Binding of CypA to CD147 activates the mitogen-activated protein kinase (MAPK) signaling pathway, which contributes to cancer cell proliferation and migration [21]. To determine whether CypA is a key molecular target of compound 9 in GC cells, we investigated the effect of compound 9 on CD147 expression and MAPK signaling in AGS cells. Treatment with compound 9 inhibited the expression of CD147 in a dose-dependent manner (Figure 3). In addition, compound 9 suppressed the phosphorylation of c-Jun N-terminal kinase (JNK) and extracellular signal regulated protein kinase 1/2 (ERK1/2) without affecting the total protein levels. However, p38 MAPK phosphorylation showed a biphasic response based on the concentration of compound 9. These results suggest that compound 9 may downregulate CD147-mediated MAPK signaling pathway, including JNK and ERK1/2, by inhibiting the expression of CypA and CD147.

### 2.3. CypA Silencing Inhibits Proliferation, Migration, and Invasion of AGS Cells 

**Figure 4:**
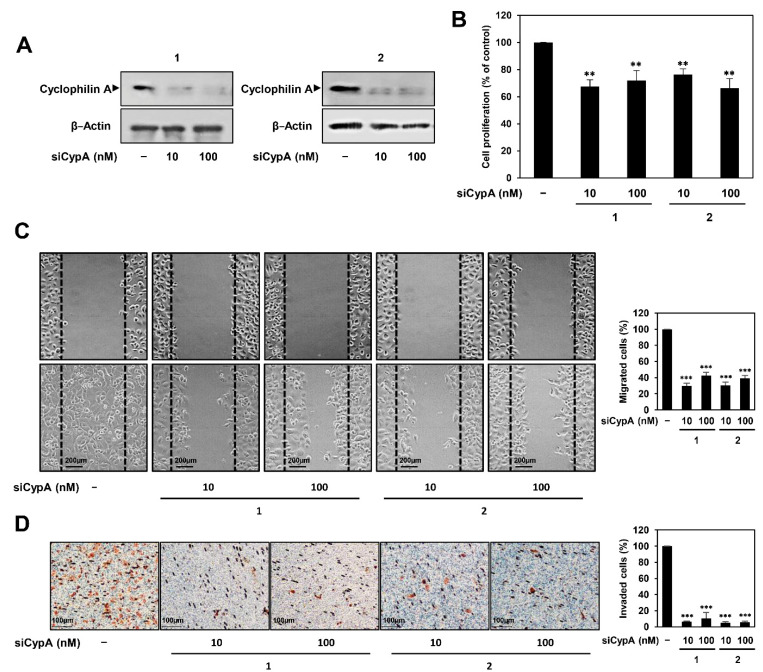
Effect of CypA knockdown on the proliferation, migration, and invasion of AGS cells. AGS cells were transfected with two types of CypA siRNAs (10 and 100 nM). (**A**) Knockdown of CypA gene was confirmed by western blot analysis. (**B**–**D**) Effect of CypA knockdown on the (**B**) proliferation, (**C**) migration, and (**D**) invasion of AGS cells. ** *p* < 0.01, *** *p* < 0.001 vs. the control.

In our previous study, we found that compound 9 effectively suppressed the proliferation, migration, and invasion of AGS cells [14]. To further verify that CypA is a biologically relevant target of compound 9, we performed a CypA genetic knockdown experiment using RNA interference in AGS cells and analyzed the resulting phenotypes. AGS cells were transfected with two types of human CypA-specific siRNAs (siCypA 1 and 2), and CypA silencing was confirmed by western blotting (Figure 4A). We first investigated the effect of CypA knockdown on the proliferation of AGS cells. Cell proliferation was significantly inhibited following CypA knockdown (Figure 4B). Furthermore, CypA knockdown effectively blocked the migration and invasion of AGS cells (Figure 4C,D). These results demonstrate that there is a significant phenotypic correlation between compound 9 and CypA silencing.

### 2.4. CypA Silencing Downregulates CD147-Mediated MAPK Signaling Pathway in AGS Cells

**Figure 5:**
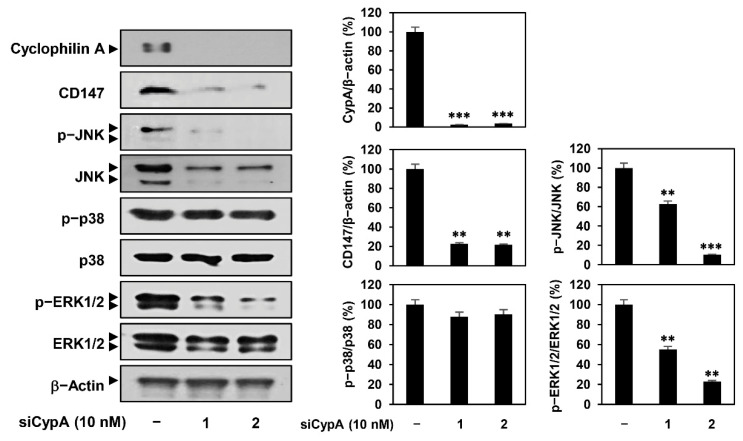
Effect of CypA knockdown on the CD147-mediated MAPK signaling pathway in AGS cells. AGS cells were transfected with CypA siRNAs (10 nM) and incubated for 24 h. Protein levels were detected by western blot analysis and further quantified by densitometry. The level of β-actin was used as an internal control. ** *p* < 0.01, *** *p* < 0.001 vs. the control.

Next, we investigated whether CypA knockdown affected CD147 expression and its downstream MAPK signaling in AGS cells. As shown in Figure 5, CD147 expression was markedly reduced by siCypA 1 and 2. Moreover, the phosphorylation of JNK and ERK1/2 was suppressed by CypA knockdown, whereas that of p38 MAPK was not. These data indicate that CypA silencing leads to the downregulation of the CD147-mediated MAPK signaling pathway in AGS cells, similar to compound 9 treatment.

### 2.5. CypA Silencing Induces Cell Cycle Arrest and Apoptosis of AGS Cells

**Figure 6:**
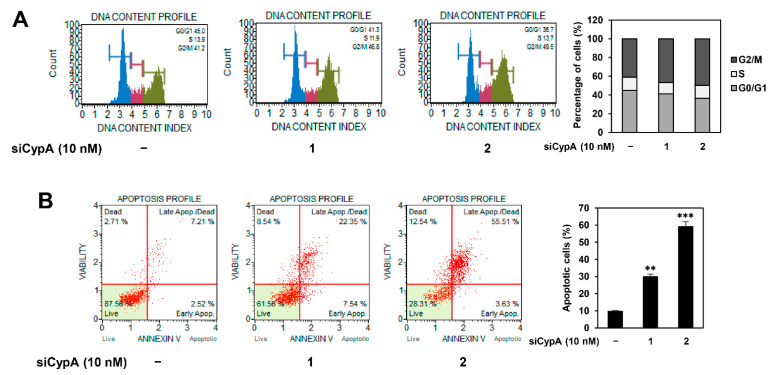
Effect of CypA knockdown on the cell cycle progression and apoptosis of AGS cells. AGS cells were transfected with CypA siRNAs (10 nM) and incubated for 72 h. (**A**) The cell cycle distribution and (**B**) apoptotic cell death were analyzed using a Muse cell analyzer and Muse analysis software. ** *p* < 0.01, *** *p* < 0.001 vs. the control.

Our previous study revealed that compound 9 inhibits the growth of AGS cells by inducing G2/M cell cycle arrest and caspase-mediated apoptosis through the downregulation of the PI3K/AKT/mTOR pathway [14]. To determine whether these effects of compound 9 are linked to the regulation of CypA function, we assessed the effect of CypA knockdown on cell cycle distribution and apoptosis of AGS cells using flow cytometry. As shown in Figure 6A, both siCypA 1 and 2 caused a decrease in the cell population at the G0/G1 phase, whereas it caused an increase in the G2/M phase cell population compared to that in control cells. In addition, both the CypA siRNAs elevated the apoptotic cell population compared to that in the control cells (Figure 6B). These results demonstrate that CypA silencing triggers G2/M cell cycle arrest and apoptosis in AGS GC cells, similar to the effect of compound 9 treatment.

**Figure 7:**
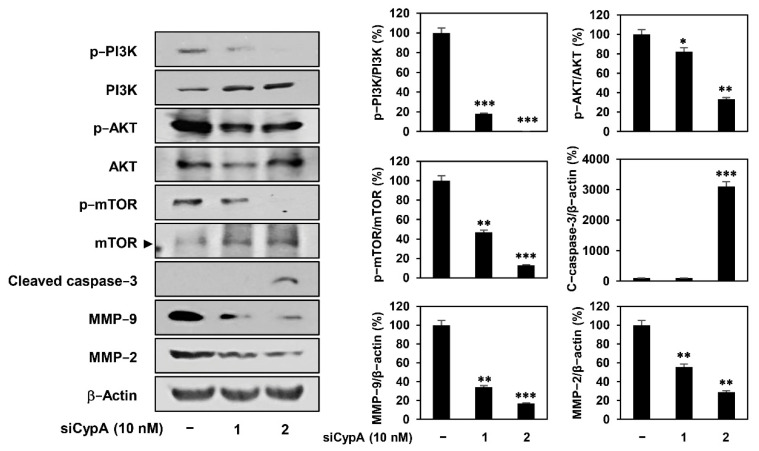
Effect of CypA knockdown on the PI3K/AKT/mTOR pathway in AGS cells. AGS cells were transfected with CypA siRNAs (10 nM) and incubated for 24 h. Protein levels were detected by western blot analysis and further quantified by densitometry. The level of β-actin was used as an internal control. * *p* < 0.05, ** *p* < 0.01, *** *p* < 0.001 versus the control.

Next, we evaluated the effect of CypA knockdown on the PI3K/AKT/mTOR pathway in AGS cells. As shown in Figure 7, CypA knockdown inhibited the phosphorylation of PI3K, AKT, and mTOR without significantly affecting the total protein levels. Furthermore, CypA knockdown activated caspase-3, a critical executioner of apoptosis, and suppressed the expression of matrix metalloproteinase (MMP)-9 and MMP-2 that play an important role in tumor invasion and metastasis, consistent with the effect of compound 9 treatment in our previous study (Figure 7). Taken together, our results suggest that the anticancer activity of compound 9 against AGS cells is closely related to the downregulation of CypA function.

### 2.6. CypA Silencing Inhibits AGS Cell-Induced Angiogenesis of HUVECs

**Figure 8:**
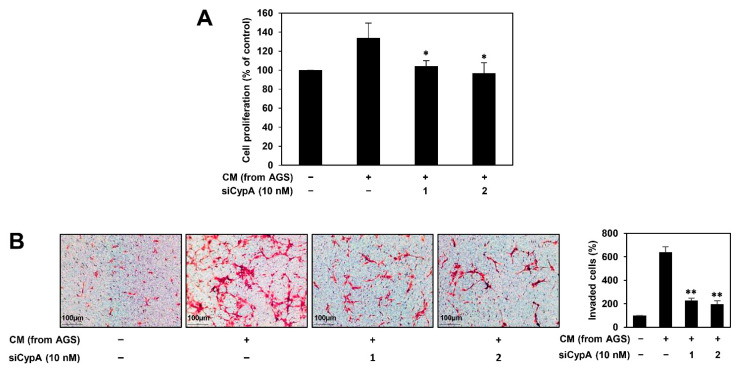
Effect of CypA knockdown on the AGS cell-induced angiogenesis of HUVECs. AGS cells were transfected with CypA siRNAs (10 nM) and incubated for 24 h. CM was obtained from the transfected cells and used for the (**A**) proliferation and (**B**) invasion of HUVECs. * *p* < 0.05, ** *p* < 0.01 vs. the control.

We previously found that compound 9 decreased in vitro AGS cell-induced angiogenesis of HUVECs by blocking the expression of HIF-1α and VEGF in AGS cells [15]. To assess whether CypA knockdown affects the angiogenic properties of HUVECs induced by AGS cells, conditioned medium (CM) was obtained from AGS cells and used as an angiogenic stimulus for the proliferation and invasion of HUVECs. As shown in Figure 8A, the proliferation of HUVECs was significantly induced by culture in CM from AGS cells compared to that in control (medium only). However, CM from CypA-deficient AGS cells inhibited the proliferation of HUVECs. In addition, the invasion of HUVECs promoted by CM from AGS cells was markedly suppressed by CypA knockdown (Figure 8B). These data indicate that CypA silencing diminishes the angiogenesis-promoting ability of AGS cells. 

**Figure 9:**
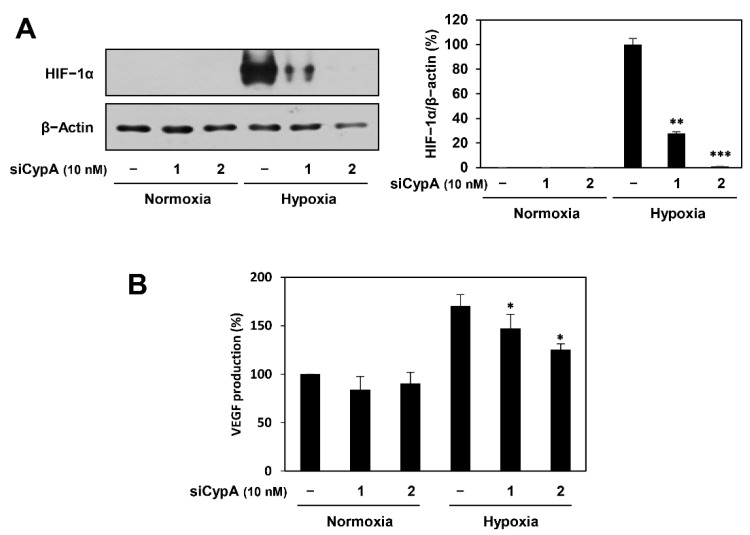
Effect of CypA knockdown on the HIF-1α and VEGF expression in AGS cells. AGS cells were transfected with CypA siRNAs (10 nM) and incubated for 24 h. The cells were incubated for 8 h under either normoxic or hypoxic conditions. (**A**) HIF-1α protein levels were detected by western blot analysis and further quantified by densitometry. The level of β-actin was used as an internal control. (**B**) The concentration of VEGF protein in the supernatant was determined by a VEGF ELISA. * *p* < 0.05, ** *p* < 0.01, *** *p* < 0.001 vs. the hypoxic control.

Next, we examined the effect of CypA knockdown on HIF-1α and VEGF expression in AGS cells. HIF-1α plays a critical role in promoting tumor angiogenesis by activating the transcription of major proangiogenic factors, including VEGF [22]. As shown in Figure 9A,B, CypA knockdown significantly decreased the accumulation of HIF-1α protein as well as the production of VEGF induced by hypoxia in AGS cells. Therefore, the inhibitory activity of CypA silencing on AGS cell-induced angiogenesis may be associated with the downregulation of HIF-1α and VEGF expression. These results imply that CypA is a cellular target protein responsible for the antiangiogenic potential of compound 9 in GC cells.

### 2.7. Identification of Binding between Compound 9 and CypA using DARTS and CETSA

**Figure 10:**
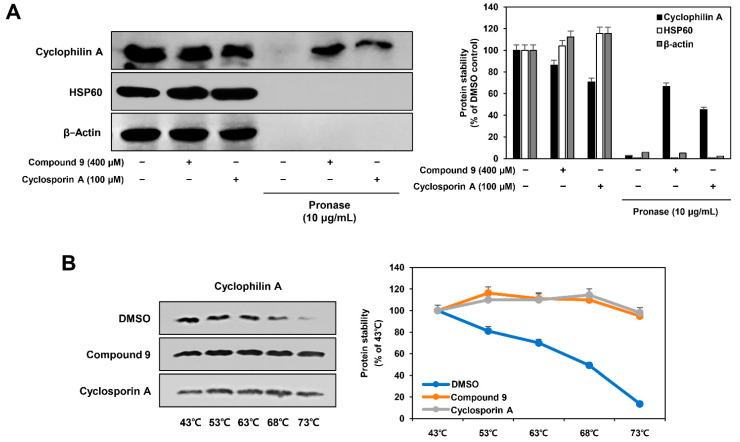
Identification of binding between compound 9 and CypA using (**A**) DARTS and (**B**) CETSA. (**A**) AGS cell lysates were incubated with DMSO, compound 9 (400 µM), or cyclosporin A (100 µM) for 1 h and then treated with pronase for 1 h. Protein levels were detected by western blot analysis and further quantified by densitometry. (**B**) AGS cell lysates were incubated with DMSO, compound 9 (400 µM), or cyclosporin A (100 µM) for 1 h and then heated at different temperatures (43–73 °C) for 5 min. The supernatants of the heated samples were analyzed by western blotting and further quantified by densitometry.

Our current results show that compound 9 mediates anticancer and antiangiogenic activities by downregulating the function of CypA in AGS cells. We further investigated whether compound 9 bound to CypA using DARTS analysis and western blotting. The DARTS approach has been successfully utilized to identify the binding of compounds to proteins of interest [23]. When a compound is incubated with protein lysate, it binds to target proteins and protects them from degradation by proteases, while non-target proteins are degraded. As shown in Figure 10A, when AGS cell lysates were pretreated with vehicle control (DMSO), CypA protein was degraded by pronase. However, pretreatment with compound 9 significantly prevented CypA degradation by pronase. In addition, CypA stability was considerably increased by treatment with cyclosporin A, an immunosuppressant that is widely known to form a complex with CypA to block the phosphatase activity of calcineurin [24]. It has been reported that heat shock proteins form a chaperone complex with cyclophilins to regulate protein folding, translocation, and assembly [25,26,27]. We further assessed whether compound 9 binds to heat shock protein 60 (HSP60), because compound 9 treatment inhibited the expression of HSP60 in AGS cells as shown in Figure 2. Neither compound 9 nor cyclosporin A prevented HSP60 degradation by pronase, indicating that compound 9 does not bind to HSP60 (Figure 10A). We also confirmed that a non-binding protein, β-actin, was completely degraded by pronase, even after treatment with either compound 9 or cyclosporin A. These results imply that compound 9 specifically binds to CypA in AGS cells, similar to cyclosporin A. Therefore, the interaction between compound 9 and CypA may result in a reduction in the protein expression of HSP60, which forms a chaperone complex with CypA.

Next, to validate the specific binding of compound 9 and CypA, we performed CETSA based on ligand-induced changes in protein thermal stability [28]. A protein-ligand complex is less likely to aggregate with increasing temperature, compared to the protein alone. As shown in Figure 10B, when AGS cell lysates were pretreated with vehicle control (DMSO), CypA protein stability decreased in a temperature-dependent manner. However, pretreatment with compound 9 stabilized CypA from protein aggregation caused by increasing the temperature. Moreover, cyclosporin A thermally stabilized its target protein, CypA. Therefore, our DARTS and CETSA data demonstrate that compound 9 binds to CypA, thereby, regulating the biological function of the target protein in AGS cells.

### 2.8. In Silico Docking Analysis of Interaction between Compound 9 and CypA

**Figure 11:**
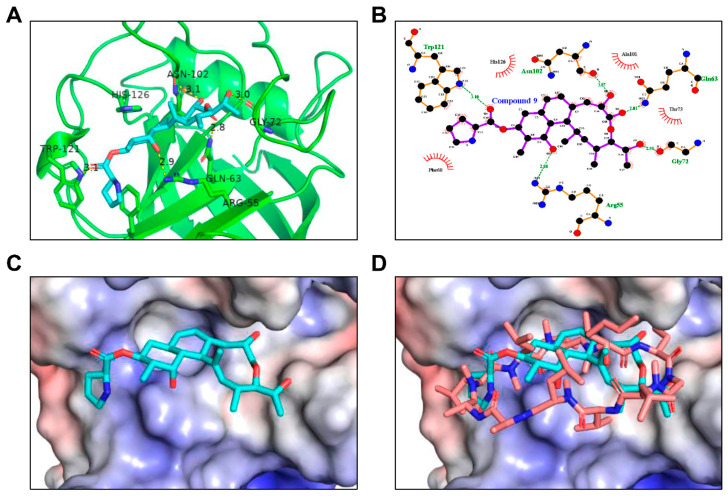
In silico docking analysis of interaction between compound 9 and CypA. (**A**–**C**) The docking model of compound 9 to CypA obtained from the AutoDock Vina 1.1.2 programs. (**D**) Comparison of the proposed binding model of compound 9 and cyclosporin A. Compound 9 is indicated in cyan and cyclosporin A in brown.

To further elucidate the binding of compound 9 to CypA at the molecular level, we simulated a binding model of CypA with compound 9 based on the crystal structure of a complex between CypA and cyclosporin A (protein data bank ID: 1CWA) and AutoDock Vina 1.1.2 programs [29]. Docking modeling revealed that compound 9 binds to CypA via hydrogen bonding interactions with Arg55, Gln63, Asn102, Gly72, and Trp121 residues and hydrophobic interactions with Phe60, Ala101, Thr73, and His126 residues of CypA (Figure 11A,B). In addition, the macrolide moiety of compound 9 snugly fits the hydrophobic pocket of CypA, and its pyrrolidine group is involved in a π-π interaction with the aromatic residue of Phe60 in CypA. By analyzing the results of the docking studies, we found that compound 9 structurally overlapped with cyclosporin A in the CypA docking model (Figure 11C,D).

## 3. Discussion

In the present study, for the first time, we identified the primary molecular target of compound 9 in GC cells through chemical proteomic analysis using 2-DE coupled with MALDI-TOF MS. Among the ten proteins downregulated more than 3-fold by compound 9 in AGS cells, CypA was validated as the protein with the most significant change by western blot analysis. In addition, we confirmed the specific interaction between compound 9 and CypA using the DARTS and CETSA methods. Furthermore, silencing of CypA using siRNA revealed that the anticancer and antiangiogenic activities of compound 9 against AGS cells were associated with the downregulation of CypA function. These results suggest that compound 9 is a new small molecule that targets CypA in GC cells.

CypA is a member of the immunophilin family and was originally identified as an intracellular binding target for cyclosporin A [24]. It is a peptidyl prolyl cis-trans isomerase that plays an important role in protein folding [30]. Intracellular CypA is secreted to the outside of cells, and the secreted CypA regulates multiple cell signaling cascades through its cellular receptor, CD147 [19]. CD147, also known as extracellular matrix metalloproteinase inducer (EMMPRIN), is a ubiquitously distributed transmembrane glycoprotein belonging to the immunoglobulin superfamily [20]. It has implicated in many physiological and pathological processes through interactions with different binding partners, including cyclophilins and integrins [31]. Several studies have demonstrated overexpression of CypA and CD147 in many malignant tumors, such as breast cancer, lung cancer, pancreatic cancer, GC, and melanoma [32,33,34,35,36]. CypA/CD147 interaction promotes tumor proliferation, invasion, metastasis, and tumor angiogenesis, and contributes to chemoresistance and cancer recurrence by mediating the activation of complex oncogenic signaling pathways, including PI3K and MAPK [37,38]. Therefore, CypA and CD147 have been recognized as potential therapeutic targets for cancer. 

Recent studies have suggested the association of CypA and CD147 with GC proliferation, invasion, metastasis, and recurrence [32,39,40]. The elevated expression of CypA was observed in GC tissues compared with normal gastric mucosa [32]. In addition, the suppression of CypA expression reduced the proliferation of BGC-823 and SGC-7901 GC cell lines through downregulating the ERK1/2 signaling pathway, as well as inhibited the tumor growth in BGC-823 GC cell line-derived xenograft mouse model [32]. Meanwhile, upregulated expression of CD147 enhanced invasion and angiogenesis via increasing MMP and VEGF expression of GC cells [39]. Moreover, Kaplan-Meier analysis revealed significantly worse disease-free and overall survival for GC patients with CD147 positive tumors, indicating that CD147 is a marker of tumor recurrence and prognosis in GC [40]. However, the role of CypA in regulating CD147 function in GC has not yet been fully elucidated. Previous reports have revealed that cyclophilins regulate the cell surface expression of several proteins, such as insulin receptor, Flt3 ligand, and CD147 [41,42,43]. In all these cases, cyclosporin A reduced the cell surface expression of the proteins. Our results showed that compound 9 inhibited the expression of CypA and CD147, resulting in the downregulation of the MAPK signaling pathway, including JNK and ERK1/2, in AGS cells. Similar to compound 9 treatment, CypA silencing reduced the expression of CD147 and the phosphorylation of JNK and ERK1/2. Furthermore, the suppressive effects of CypA silencing on the proliferation, migration, invasion, and angiogenesis induction of AGS cells were associated with G2/M cell cycle arrest, caspase-mediated apoptosis, inhibition of MMP-9 and MMP-2 expression, inactivation of PI3K/AKT/mTOR pathway, and inhibition of HIF-1α and VEGF expression, consistent with the effects of compound 9 reported in our previous studies [14,15]. Taken together, our findings imply that CypA regulates CD147 expression in GC, and thus, it affects CD147-mediated downstream signaling and subsequent cellular response. Accordingly, compound 9 may interfere with the function of CD147 in activating growth, metastasis, and angiogenesis in GC cells by targeting CypA.

In this study, the binding mode of compound 9 to CypA was further investigated using a docking modeling analysis. Our results revealed that the structure of compound 9 was a good fit for the cyclosporin A binding cavity of CypA. Three-dimensional structure analysis of the binding between two molecules will help to decipher the exact binding mechanism of compound 9 to CypA. However, compound 9 exhibited greater in vitro anticancer activity against AGS cells at higher concentrations compared to cyclosporin A, indicating the existence of unidentified interacting proteins of compound 9 that may affect its anticancer effects. For this reason, we need to identify other binding proteins and the related molecular mechanisms of compound 9 for lead optimization. Moreover, in vivo studies using animal models are necessary to clarify the effectiveness and safety of compound 9, because cyclosporin A has several side effects, including kidney damage, high blood pressure, infection, and headache [44]. In conclusion, our findings demonstrate that CypA is a cellular target protein of compound 9 and implicate the critical role of CypA/CD147 interaction in GC. Therefore, CypA may be a potential therapeutic target for GC, and compound 9 may be a promising novel agent for the treatment of GC.

## 4. Materials and Methods

### 4.1. Materials

Compound 9 was isolated from the culture extract of *Nocardia* sp. CS682 mutant, as shown in our previous report [14], and was prepared at a concentration of 100 mM using dimethyl sulfoxide (DMSO). Endothelial growth medium-2 (EGM-2) and antibiotics were purchased from Lonza (Walkersville, MD, USA), and fetal bovine serum (FBS) and RPMI-1640 medium were purchased from Invitrogen (Grand Island, NY, USA). Transwell chamber system, VEGF enzyme-linked immunosorbent assay (ELISA) kit, and Matrigel were purchased from Corning Costar (Acton, MA, USA), Koma Biotech (Seoul, Korea), and BD Biosciences (San Jose, CA, USA), respectively. Cyclosporin A, gelatin, and 3-[4,5-dimethylthiazol-2-yl]2,5-diphenyl tetrazolium bromide (MTT) were purchased from Sigma-Aldrich (St. Louis, MO, USA). The Muse™ Annexin V and dead cell assay kit (cat. no. MCH100105), and Muse Cell Cycle Kit (cat. no. MCH100106) were purchased from Merck Millipore (Darmstadt, Germany). Antibodies against HSP60 (60 kDa; cat. no. 12165), HP1-gamma (22 kDa; cat. no. 2619), annexin 7 (51 kDa; cat. no. 3666), cyclophilin A (18 kDa; cat. no. 2175), CD147 (58 kDa; cat. no. 13287), phospho-JNK (Thr183/Tyr185, 46/54 kDa; cat. no. 9251), JNK (46/54 kDa; cat. no. 9252), phospho-p38 (Thr180/Tyr182, 43 kDa; cat. no. 4511), p38 (40 kDa; cat. no. 8690), phospho-ERK1/2 (Thr202/Tyr204, 42,44 kDa; cat. no. 9101), ERK1/2 (42,44 kDa; cat. no. 9102), phospho-PI3K (Tyr458, 85 kDa; cat. no. 4228), PI3K (85 kDa; cat. no. 4257), phospho-AKT (Ser473, 60 kDa; cat. no. 4060), AKT (60 kDa; cat. no. 9272), phospho-mTOR (Ser2448, 289 kDa; cat. no. 2971), mTOR (289 kDa; cat. no. 2972), cleaved caspase-3 (17 kDa; cat. no. 9661), MMP-9 (92 kDa; cat. no. 3852), MMP-2 (72 kDa; cat. no. 4022), rabbit IgG (cat. no. 7074), and mouse IgG (cat. no. 7076) were purchased from Cell Signaling Technology (Danvers, MA, USA). Antibodies against HIF-1α (120 kDa; cat. no. 610959), and β-actin (42 kDa; cat. no. ab6276) were purchased from BD Biosciences (Franklin Lakes, NJ, USA) and Abcam (Cambridge, UK), respectively.

### 4.2. Cell Culture and Hypoxic Conditions

Human GC AGS cells and HUVECs were obtained from the Korean Cell Line Bank (Seoul, Korea) and American Type Culture Collection (Manassas, VA, USA), respectively. AGS cells and HUVECs were grown in RPMI and EGM-2 medium supplemented with 10% FBS. The cells were maintained at 37 °C in a humidified 5% CO_2_ incubator (Thermo Scientific, Vantaa, Finland). For hypoxic conditions, the cells were incubated in a hypoxic chamber (SANYO, Chuo-ku, Osaka, Japan) under 5% CO_2_ and 1% O_2_ with balance N_2_ calibration gas.

### 4.3. Preparation of Total Cellular Extract

Untreated and compound 9-treated AGS cells (2 × 10^7^ cells) were collected, washed with cold phosphate-buffered saline (PBS), re-suspended in 200 μL of lysis buffer, and incubated at 4 °C for 1 h. The cells were lysed by sonication and centrifuged to collect the supernatant. Protein concentration was determined using the Bradford protein assay, according to the manufacturer’s instructions.

### 4.4. Two-Dimensional Gel Electrophoresis (2-DE) and Image Analysis

Protein samples (1.5 mg) were applied to immobilized pH gradient (IPG) strips (17 cm, pH 3–10 NL, Bio-Rad Laboratories, Hercules, CA, USA) using a passive rehydration method. The second dimension was performed using 12% sodium dodecyl sulfate-polyacrylamide gel electrophoresis (SDS-PAGE) at a constant current of 40 mA per gel after isoelectric focusing (IEF) and equilibration. The gels were stained using Coomassie brilliant blue (CBB) R-250 staining solution (Bio-Rad) and scanned using a gel documentation system (Bio-Rad). The image analysis, including image editing, spot finding, quantitation, and matching, were performed using the two-dimensional analysis software package Delta2D 3.6/DECODON (DECODON GmbH, Greifswald, Germany). The software was used to calculate spot intensity over the spot area.

### 4.5. Matrix Assisted Laser Desorption Ionization-Time of Flight/Mass Spectrometry (MALDI-TOF/MS) Analysis and Protein Identification

Selected protein spots in the gel were excised and destained with destaining solution for 5 min. The gel pieces were washed with water, 50 mM ammonium bicarbonate, and 50% acetonitrile for 30 min. The gel pieces were dehydrated with 100% acetonitrile and dried. The dried gel pieces were then incubated with 40 μL of 50 mM ammonium bicarbonate containing 20 ng of trypsin (Promega, Madison, WI, USA) overnight at 37 °C. The samples were desalted using a C18 ziptip (Millipore) and then eluted with a matrix (Agilent, Palo Alto, CA, USA) for MALDI-TOF/MS analysis. Protein identification was performed by searching MS/MS spectra of a subset of peptides against the Swiss-Prot database using MASCOT.

### 4.6. Western Blot Analysis

Following treatment, the cells were collected and lysed in RIPA buffer (ATTO, Tokyo, Japan) supplemented with a protease inhibitor cocktail (Roche Diagnostics, Indianapolis, IN, USA) on ice. Equal amounts of lysates were separated by 10% SDS-PAGE. The separated proteins were then transferred to polyvinylidene difluoride (PVDF) membranes (EMD Millipore, Hayward, CA, USA) and blocked using Tris-buffered saline with Tween-20 (TBST) containing 5% skim milk at room temperature for 1 h. The membranes were then incubated with primary antibodies against HSP60 (1:2000), HP1-gamma (1:2000), annexin 7 (1:2000), cyclophilin A (1:2000), CD147 (1:2000), phospho-JNK (1:2000), JNK (1:2000), phospho-p38 (1:2000), p38 (1:2000), phospho-ERK1/2 (1:2000), ERK1/2 (1:2000), phospho-PI3K (1:2000), PI3K (1:2000), phospho-AKT (1:2000), AKT (1:2000), phospho-mTOR (1:2000), mTOR (1:2000), cleaved caspase-3 (1:2000), MMP-9 (1:2000), MMP-2 (1:2000), HIF-1α (1:2000), and β-actin (1:10000) overnight at 4 °C. After washing with TBST three times, the membranes were incubated with horseradish peroxidase-conjugated anti-rabbit (1:3000) or anti-mouse (1:3000) secondary antibody for 1 h at room temperature. Immunolabeling was detected using an enhanced chemiluminescence (ECL) kit (Bio-Rad Laboratories) according to the manufacturer’s instructions. The band density was analyzed using ImageJ software (version 1.5; NIH).

### 4.7. CypA-Directed RNA Interference

To knockdown the expression of CypA, two small interfering RNAs (siRNAs) for CypA were obtained from Bioneer (Daejeon, Korea), and the sequences were as follows: siRNA#1 (sense) 5′-AAGAUGAGAACUUCAUCCU-3′; (antisense) 5′-AGGAUGAAGUUCUCAUCUU-3′; siRNA#2 (sense) 5′-GCUCGCAGUAUCCUAGAAU-3′; (antisense) 5′-AUUCUAGGAUACUGCGAGC-3′. Cells were transfected with siRNAs (10 and 100 nM) in serum-free medium using Lipofectamine^TM^ 3000 Reagent (Invitrogen, NY, USA). CypA expression was determined using western blot analysis.

### 4.8. Cell Proliferation Assay

Cells (2 × 10^4^ cells/well) were plated in a 48-well culture plate and transfected with siRNAs (10 and 100 nM). After incubation for 72 h, cell proliferation was measured using the 3-(4,5-dimethylthiazol-2-yl)-2,5-diphenyltetrazolium bromide (MTT) colorimetric assay at 540 nm.

### 4.9. Wound Healing Assay

Cells (15 × 10^4^ cells/well) were plated in a 24-well culture plate. After incubation for 24 h, the cells were scratched using a 10 μL of pipette tip, washed with PBS, and transfected with siRNAs (10 and 100 nM). After incubation for 24 h, images were obtained using a 100× optical microscope (Olympus, Tokyo, Japan).

### 4.10. Invasion Assay

Cell invasiveness was investigated using a Transwell chamber system with polycarbonate filter inserts with a pore size of 8.0 µm. The lower surface of the filter was coated with 10 µL of gelatin (1 mg/mL) for 1 h, and the upper surface was coated with 10 µL of Matrigel (3 mg/mL) for 1 h. The transfected cells (4 × 10^4^ cells/well) were plated in the upper chamber of the filter. The chamber was incubated for 18 h, and then the cells were fixed with 70% methanol and stained with hematoxylin and eosin (H&E) at room temperature for 5 min. The total invaded cells were photographed in randomly selected fields using an optical microscope (Olympus) at 200× magnification.

### 4.11. Cell Cycle Analysis

Cell cycle analysis was performed using a Muse™ cell cycle kit, according to the manufacturer’s instructions. Cells (2 × 10^5^ cells/well) were plated in a 6-well culture plate and transfected with siRNAs (10 nM). After 72-h of incubation, the cells were collected, washed with PBS, and fixed with cold 70% ethanol. After overnight storage at -20 °C, ethanol was removed, and the cells were washed with PBS. Further, 200 μL of Muse cell cycle reagent was added and reacted in the dark for 30 min. The percentage of cells in G0/G1, S, and G2/M phases was then calculated using the Muse cell analyzer and Muse analysis software (MuseSoft_V1.8.0.3; Luminex Corporation, Austin, TX, USA). The Muse cell cycle software module displays the data in two plots: a dot plot displaying DNA Content Index versus Cell Size Index for setting the gate and a histogram displaying DNA Content Index versus Count for assessing the percentage of cells in each phase.

### 4.12. Apoptosis Analysis

Apoptosis analysis was performed using a Muse™ Annexin V and dead cell assay kit according to the manufacturer’s instructions. Cells (2 × 10^5^ cells/well) were plated in a 6-well culture plate and transfected with siRNAs (10 nM). After a 72-h incubation, the cells were collected, washed with PBS, and stained with Muse apoptosis reagents in the dark for 20 min. The stained cells were analyzed using a Muse cell analyzer and Muse analysis software.

### 4.13. Tumor Cell-Induced Proliferation Assay

AGS cells (1 × 10^5^ cells/well) were plated in a 24-well culture plate and transfected with siRNAs (10 nM). After incubation for 24 h, the medium was replaced with fresh medium. After further incubation for 24 h, CM was obtained from the AGS cells and used as the angiogenic stimulus for the proliferation of HUVECs. After incubation for 72 h, cell proliferation was measured using MTT assay.

### 4.14. Tumor Cell-Induced Invasion Assay

AGS cells (1 × 10^5^ cells/well) were plated in the lower chamber and transfected with siRNAs (10 nM). After 24 h of incubation, the medium in the lower chamber was replaced with fresh medium. Serum-starved HUVECs (6 × 10^4^ cells/well) were placed in the upper chamber and incubated for 18 h. The HUVECs that invaded the lower chamber were analyzed using the same procedure used for the invasion assay.

### 4.15. Measurement of VEGF by ELISA

VEGF concentration in AGS cells was measured using a VEGF immunoassay kit (Koma Biotech, Seoul, Korea). Cells (1 × 10^5^ cells/well) were plated in a 24-well culture plate and transfected with siRNAs (10 nM). After 24 h of incubation, the cells were incubated for 8 h under either normoxic or hypoxic conditions. The supernatants were collected, and VEGF protein levels were measured according to the manufacturer’s instructions.

### 4.16. DARTS Assay

Cells were collected and lysed using RIPA buffer (ATTO). Protein samples (99 µL, 2 mg/mL) were incubated with 1 µL of DMSO, compound 9 (400 µM), or cyclosporin A (100 µM) in the dark at 4 °C for 1 h. The samples were then treated with pronase (10 μg/mL, Roche, Basal, Switzerland) for proteolysis in the dark at room temperature for 1 h. After digestion, the samples were incubated with protease inhibitor (Roche) at 4 °C for 10 min, mixed with 6x SDS sample buffer, and analyzed by SDS-PAGE and western blotting.

### 4.17. CETSA

Cells were collected and lysed using RIPA buffer (ATTO). Protein samples (99 µL, 2 mg/mL) were incubated with 1 µL of DMSO, compound 9 (400 µM), or cyclosporin A (100 µM) in the dark at 4 °C for 1 h. The samples were heated at different temperatures for 5 min in a heat block (Benchmark Scientific, NJ, USA), followed by cooling for 5 min at room temperature. The heated samples were centrifuged at 20,000× *g* for 20 min at 4 °C, and the supernatants were mixed with 6xSDS sample buffer and analyzed by SDS-PAGE and western blotting.

### 4.18. Ligand/Molecular Target Selection and Preparation

Two-dimensional structures of the ligand for the study were obtained using MarvinSketch (Version 5.11, academic package, ChemAxon). Three-dimensional structure coordinates were generated and preliminarily optimized using the MMFF94 algorithm implemented in MarvinSketch. All ligands were searched in the protein data bank (PDB), and the obtained PDB-files were converted into PDBQT format (input for AutoDock Vina). The charges on ligand atoms were generated using the Gastieger model, nonpolar hydrogen was merged, and default retaining bonds were retained using the TORSDOF utility in AutoDockTools version 1.5.6 (http://mgltools.scripps.edu, accessed on 20 November 2020).

The crystal structure of the protein (monomeric cyclophilin A in complex with cyclosporin A and the natural ligand [cyclosporin A], PDB ID:1CWA) resolved at 2.10 Å was retrieved from the PDB (http://www.rcsb.org, accessed on 20 November 2020). To eliminate the collision of amino acid residues and ligands in a new environment, some of the ligands in the structure were removed using PyMol tools version 2.4.1. Following the removal, the resulting structure was saved as a pdb file, and the charge and hydrogen were then added using AutoDockTools software. The optimized structure was saved as a pdbqt file. The grid box was set to be located in the center of the original structure of the cyclosporin A structure, and the grid box size was set to 20 × 24 × 20 Å^3^, spacing 1 Å, and exhaustiveness 8.

### 4.19. In Silico Docking Analysis

The docking simulation was performed using AutoDock Vina 1.1.2. The active site of the protein structure was kept rigid, and inflexible docking was performed. The parameters were set to exhaustiveness 8, num_modes 10, and energy_range 3 kcal/mol. The Vina code predicts the adopted form with a binding affinity (kcal/mol). The best docking pose was analyzed according to the binding affinity obtained from 50 independent runs to create the final docked pose. The binding energy of a cluster is the average binding energy of all the conformations in it. The clusters with the lowest binding energy and the highest number of conformational structures were chosen as representative binding modes for the ligand. The protein-ligand complexes were visualized using PyMol Version 2.4.1 (Schrödinger, New York, NY, USA, https://pymol.org/, accessed on 20 November 2020) and LigPlot+ version 2.2.4.

### 4.20. Statistical Analysis

The results are presented as mean ± standard deviation (SD) of data from experiments performed in triplicate. All the statistical analyses were carried out using Microsoft Excel 2017 (Albuquerque, NM, USA). If the data passed the tests for normality and homogeneity of variance, a one-way analysis of variance (ANOVA) followed by Tukey’s test as post hoc analysis was used to compare the different groups. SPSS statistics package (SPSS 9.0; SPSS Inc., Chicago, IL, USA) was used to analyze the data, and a *p*-value of < 0.05 was considered to be a statistically significant difference and indicated by asterisk (*). Detailed information is described in each figure legends.

## 5. Conclusions

Using 2-DE and MALDI-TOF MS analysis, we identified CypA as a crucial anticancer target of compound 9 in AGS cells for the first time. Specific interaction between compound 9 and CypA was confirmed through DARTS, CETSA, and in silico docking analysis. Compound 9 downregulated CD147-mediated MAPK signaling pathway, including JNK and ERK1/2, by inhibiting CypA and CD147 expression. In addition, silencing of CypA using RNA interference revealed that it plays a critical role in the molecular mechanism that regulates the proliferation, migration, invasion, and angiogenesis of AGS cells. These findings provide new insights into the mechanisms underlying the anticancer and antiangiogenic activities of compound 9 in GC cells.

## Figures and Tables

**Table 1:** Ten protein spots identified by MALDI-TOF MS.

Spot no.	Uniprot ID	Protein Name	Accession no.	Sequence Coverage (%)/ Peptides Matched	Mascot Score	Protein MW (kDa)	pI Value	Downregulation by Compound 9 (Fold)
271	MIC60_HUMAN	motor protein	BAA04656	27/12	211	83.678	6.08	3.5
516	CH60A_ARATH	heat shock protein 60 (HSP60)	NP_189041	19/17	249	61.281	5.7	5.3
736	ANXA7_HUMAN	synexin (Annexin VII)	AAA36616	17/11	525	50.316	6.25	3.9
1165	MLEC_HUMAN	Malectin	Q14165	22/10	128	32.234	5.27	5.2
1216	CAPZB_HUMAN	F-actin capping protein beta subunit	AAA87395	15/14	226	30.629	5.69	5.3
1257	LEG3_HUMAN	IgE-binding protein	AAA35607	26/14	200	26.188	8.58	3.0
1541	CBX3_HUMAN	HP1-gamma (CBX3)	AAB48101	26/20	344	19.72	5.03	3.1
1687	MYL6_HUMAN	non-muscle myosin light chain	AAA59893	21/16	105	16.961	4.46	3.7
1699	PPIA_HUMAN	peptidylprolyl isomerase A (Cyclophilin A)	CAA37039	27/23	402	18.013	7.68	3.2
1703	ATPD_HUMAN	H(+)-transporting ATP synthase	CAA45016	11/9	117	17.49	5.38	3.8

## Data Availability

The data that support the findings of this study are available from the corresponding author upon reasonable request.
